# A PetriNet-Based Approach for Supporting Traceability in Cyber-Physical Manufacturing Systems

**DOI:** 10.3390/s16030382

**Published:** 2016-03-17

**Authors:** Jiwei Huang, Yeping Zhu, Bo Cheng, Chuang Lin, Junliang Chen

**Affiliations:** 1 State Key Laboratory of Networking and Switching Technology, Beijing University of Posts and Telecommunications, Beijing 100876, China; chengbo@bupt.edu.cn (B.C.); chjl@bupt.edu.cn (J.C.); 2 Agricultural Information Institute, Chinese Academy of Agricultural Sciences, Beijing 100081, China; zhuyeping@caas.cn; 3 Department of Computer Science and Technology, Tsinghua University, Beijing 100084, China; chlin@tsinghua.edu.cn

**Keywords:** traceability, Petri net, cyber-physical manufacturing systems, product quality control

## Abstract

With the growing popularity of complex dynamic activities in manufacturing processes, traceability of the entire life of every product has drawn significant attention especially for food, clinical materials, and similar items. This paper studies the traceability issue in cyber-physical manufacturing systems from a theoretical viewpoint. Petri net models are generalized for formulating dynamic manufacturing processes, based on which a detailed approach for enabling traceability analysis is presented. Models as well as algorithms are carefully designed, which can trace back the lifecycle of a possibly contaminated item. A practical prototype system for supporting traceability is designed, and a real-life case study of a quality control system for bee products is presented to validate the effectiveness of the approach.

## 1. Introduction

A cyber-physical system (CPS) is a system of collaborating computational elements controlling physical entities. It offers cyber capability in every physical component, a high degree of automation, and reorganizing/reconfiguring dynamics [1]. Due to its unique features, CPS has been widely used in manufacturing [2,3,4]. In a manufacturing system equipped with CPS techniques, which is called a cyber-physical manufacturing system, machines as well as human workers constitute the physical resources, and data is collected from the sensors of these resources which constitutes the cyber part of the system. Computations are carried out with the objective of maximizing the utilization of limited resources while guaranteeing the dependability of manufacturing processes, and suitable decisions are taken, based on which the physical resources are further controlled. Therefore, CPS makes a traditional fixed manufacturing process become a flexible dynamic one, which can significantly improve the efficiency of the manufactures. It has become to play an important role in the design and development of modern manufacturing systems.

With the growing number and high dynamics of complex procedures in a manufacturing activity [5], the monitoring of manufacturing processes has drawn more and more attention. Government institutions, companies, traders as well as researchers have stressed the importance of tracing the entire life of every product, especially for food, clinical materials, or similar items [6]. During the production processes, data from these processes has to be recorded; when an item were examined with any defect in the marketplace after production or in any process within production, it should be quite necessary to extract valuable information from such data supporting for back-tracing the sources of the defect. Such traceability problem has become one of the most important issues in the design, implement and management of manufacturing systems.

There have been several traceability solutions for manufactures in both industry and academia. Some of them started with the underlying hardware layer, by integrating radio frequency identification (RFID) tags and readers in different objects [7]. Furthermore, with advanced RFID techniques, networked software systems supporting traceability have been designed and implemented [8]. Meantime, others worked with the application of software engineering to industrial automation including manufacturing automation and process control, mostly aiming to reduce the cost as well as complexity of the systems [9]. Most of the existing works study the traceability problem from a technical point of view, aiming at advancing modern techniques to enable traceability of the productions. In this paper, however, such issue is studied from a very different theoretical angle. The objective is to provide modeling support for describing the detailed procedures of manufactures, based on which the entire life of every product can be fully monitored within production and precisely traced back after production. A generalized Petri net model is presented to formulate the manufacturing processes, and a traceability model is automatically built by model transformation. Model-based algorithms are proposed, enabling back-tracing to the source of an item with detailed productive data. A prototype system is designed which has been largely applied to bee products quality control in reality.

The contribution of this paper is threefold as follows.

1. The traceability issue is studied from a modeling viewpoint, aiming at proposing precise modeling approaches for enabling traceability in fully automatic manufacturing systems. Different from most of the traditional approaches which trace an item by RFID tags, this paper digs into detailed production procedures and studies several basic patterns capturing elementary aspects of atomic manufacturing process.
2. Petri net model is generalized for describing manufacturing processes, and an automatic methodology of traceability model generation is designed. Algorithms based on the model are proposed, with which all the manufacturing procedures related to the item for traceability analysis can be ordinally listed with detailed production data.
3. A prototype system supporting traceability in CPS-based manufacturing is designed. Several techniques such as data entity generation, service provisioning, and service orchestration are applied, and a real-life case study for bee products quality control is presented.

The remainder of this paper is organized as follows. Section 2 discusses the related work most pertinent to this paper. Section 3 presents Petri net models for formulating manufacturing processes, and summarizes the basic patterns of them. Section 4 proposes detailed schemes of traceability analysis by designing corresponding models as well as algorithms from the original Petri net models. Section 5 presents the design of a prototype system, and introduces a case study of a quality control system for bee products in reality. Finally, the paper is concluded in Section 6.

## 2. Related Work

### 2.1. Traceability in Manufacturing Systems

Traceability has been a critical concern for manufacturing systems, especially in food and clinical industries. It has been clearly demonstrated that the ability of systems to extract valuable data from manufacturing processes is of great importance [10], and thus advanced information technology (IT) has been widely adopted in modern manufacturing systems [11]. With the rapid growth of the complexity of manufacturing processes which lead to the costliness and inefficiency of monitoring [12], IT-based solutions show powerful strength of reducing the complexity associated with traceability by human operators [6].

Several traceability approaches started with applying RFID technology to industrial systems for tracking every individual item in a manufacturing process. Cao *et al.* [13] introduced RFID technology to automotive industry as an enabler of product lifecycle information management, and enhanced the traceability of the product throughout its value chain via automatic identification. Dai *et al.* [14] adopted RFID-enabled shop-floor manufacturing solutions across the whole operations in automotive small and medium sized enterprises, and extended the efforts of the companies in setting up and integrating manufacturing execution system and enterprise resource planning system. Furthermore, Fishkin *et al.* [7] equipped wearable gloves and bracelets with RFID techniques for detecting the interactions between users and unobtrusively tagged objects.

With RFID techniques, some approaches studied the traceability issue more systematically. With the emergence of cyber-physical systems, CPS has been widely applied for real-time processing in industrial activities [15]. Kelepouris *et al.* [8] proposed an information system architecture that supports efficient information management across the food supply chain utilizing RFID technology for small and medium enterprises. Sánchez *et al.* [6] designed a general framework for developing traceability solutions in small manufacturing companies based on cyber-physical systems. Mora-Mora *et al.* [16] proposed a distributed system equipped with RFID communication technologies for enabling traceability of the flow and movement of people in smart cities. Blackburn and Denno [17] presented a simulation platform for CPS-based industrial systems to support virtual design and verification of industrial process plant designs.

Most of the existing approaches dedicated to advancing practical techniques of sensing, data collection, data storage and database management in order to facilitate the traceability analysis in manufacturing processes. With such techniques, system engineers can manually design detailed schemes with few difficulty which have benefited a lot for the manageability of manufacturing activities and the safety of products. However, few of them have looked into the procedures of traceability analysis essentially by studying general methodologies that can be universally applied to different types of industrial systems. A general approach supporting traceability analysis in manufacturing systems is urgently required.

### 2.2. Theoretical Modeling in Cyber-Physical Manufacturing Systems

Besides technical solutions, there have been several research efforts that have extensively discussed the theoretical aspects in CPS. On one hand, some of them dedicated to design precise models in order to formulate basic activities in CPS especially for manufacturing [18]. Among them, Petri net, which allows the representation of very general discrete event systems whose operations depend on potentially complex control schemes, has been widely used in several aspects of manufacturing systems. Hu *et al.* [19] applied Petri nets to distributed control in large-scale manufacturing systems. Mitchell and Chen [20] used stochastic Petri nets for reliability analysis in CPS by formulating intrusion detection and response.

On the other hand, some researchers gave attention to some mathematical foundations of cyber-physical systems. Bogdan and Marculescu [5] studied the statistical characteristics of CPS workload, and highlighted the importance of stochastic control in CPS. Kolacinski and Loparo [21] designed a mathematical framework for stability and security analysis in cyber-physical power systems.

Many of the existing approaches have shown the advantages of theoretical research in cyber-physical manufacturing systems, such as its flexibility, wide applicability, high accuracy, *etc*. However, there are few efforts studying the traceability problem from a mathematical aspect. Theoretical frameworks as well as quantitative schemes of traceability analysis in manufacturing systems remain largely unexplored. To fill this gap, this paper starts with formulating manufacturing processes using mathematical models and designs a model-based quantitative approach to support traceability of the lifecycle for every industrial product. Considering the basic requirements of industrial manufacturing, the objective of the approach is to enable precise and efficient traceability analysis especially for cyber-physical systems in which complex non-stationary physical processes like in hydraulics systems are not involved, and theoretical optimal (stochastic) control of the system is out of the scope of this paper.

## 3. Petri Net Model for Manufacturing Processes

### 3.1. Basic Concepts of Process Petri Net

The traditional Petri net mostly focuses on the control flow of dynamic processes, studying explicit conditions under which events can be enabled. In a traceability solution for manufacturing activities, besides explicit control flow, some implicit data that closely related to the manufacturing processes has to be precisely recorded and displayed, such as the weight and volume of the items in each atomic process. Therefore, this paper generalizes Petri net with quantitative data related to the transitions.

Firstly, the basic definition of a generalized Petri net, which is called *Process Petri Net (PPN)*, is presented as follows.

**Definition 1** (Process Petri Net).  *A Process Petri Net (PPN)* Σ *for formulating a manufacturing process is defined by a 5-tuple*

$$\Sigma =\left(P,T,A,w,m_{0}\right)$$

*where*

- P is the finite set of places.
- T is the finite set of transitions.
- A⊆(P×T)∪(T×P) *is the set of arcs from places to transitions and from transitions to places.*
- $w:A\to R^{+}\cup \left\{0\right\}$ *is the weight function of the arcs.*
- $m_{0}:P\to R^{+}\cup \left\{0\right\}$ *is the initial state of the net.*

A manufacturing process can be formulated by a PPN, where the *transitions* represent the atomic manufacturing activities, while the *places* denote the products (either raw materials, work-in-progress items, or products ready for sale). The *arcs* connect places to transitions and transitions to arcs, bridging the gap between activities and their related items. The *weight* related to an arc is a nonnegative real number indicating the implicit data that closely related to the manufacturing process, which can represent either weight, volume, amount, *etc*.

In a PPN, *tokens* are assigned to places, where a token essentially indicates the fact that the product described by that place has been finished by its precursor activity. Different from traditional Petri net, data of items is also attached with tokens in the PPN model. The way in which tokens are assigned to a PPN defines a *marking*, which is formally defined as follows.

**Definition 2** (marking).  *A marking m of a PPN* $\Sigma =\left(P,T,A,w,m_{0}\right)$ *is a function* $m:P\to R^{+}\cup \left\{0\right\}$

Marking *m* defines a row vector $m=[m\left(p_{1}\right),m\left(p_{2}\right),\ldots ,m\left(p_{n}\right)]$, where the *i*-th entry indicates the data attached to the tokens in place $p_{i}$. Such marking row vector defines the *state* of a PPN at certain time point. In Definition 1, $m_{0}$ is a marking indicating the state of a PPN when it is initialized.

When drawing a PPN, the convention is to use circles to represent places and bars to represent transitions. Directed arrows connecting places and transitions represent elements of the arc set, and their weights are written accompanied by the arcs. In a PPN graph, given a certain marking *m*, tokens are indicated by dark dots in the appropriate places where $m(p_{i})\neq 0$. Example 1 illustrates the PPN model of a typical manufacturing process.

**Figure 1:**
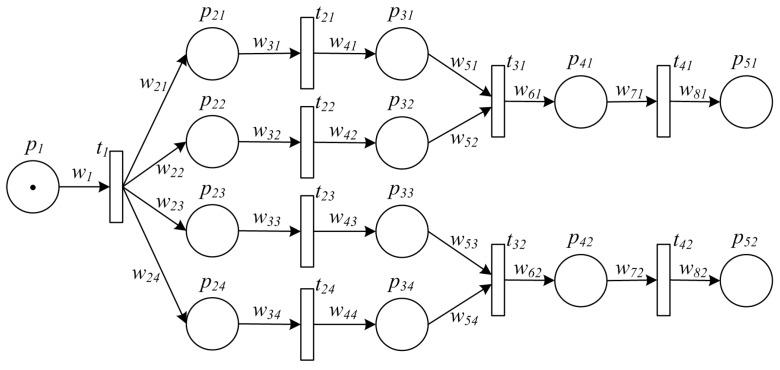
An example of PPN model for a typical manufacturing process.

**Example 1.**  *Figure 1 shows a PPN describing a typical manufacturing process, which is defined by* $\Sigma =\left(P,T,A,w,m_{0}\right)$. *Specifically, such process starts with some amount of raw material ready to proceed, whose volume is indicated by* $w_{1}$, *i.e.,* $m_{0}\left(p_{1}\right)=w_{1}$, *and* $m_{0}\left(p_{i}\right)=0$ *for* ∀i≠1. *Firstly, raw material is divided into several packages for further processing in a parallel way. On finishing all the processing, some of the intermediate products are packed together for final processing. Next, the final products are delivered to markets for sale.*

In order to use PPN to model dynamic manufacturing processes, the state transition mechanism has to be defined. Basically, for a transition t∈T to “happen”, it is required that there should be sufficient tokens in each place which is input to the transition. Formally, the notion of *enabled transition* is introduced at first as follows.

**Definition 3** (enabled transition).  *A transition* $t_{j}\in T$ *in a PPN* $\Sigma =\left(P,T,A,w,m_{0}\right)$ *is enabled under marking m if and only if (iff)*

$$m\left(p_{i}\right)\geq w\left(p_{i},t_{j}\right)>0,\;\;\;\forall p_{i}\in P$$

When a transition is enabled, we say that it can *fire*. Its formal definition can be found in Definition 4.

**Definition 4** (firing).  *When a transition* $t_{j}\in T$ *in a PPN* $\Sigma =\left(P,T,A,w,m_{0}\right)$ *is enabled under marking m, the firing of transition t is denoted by* $m\overset{t_{j}}{⟶}m^{\prime }$ *where*

$$m^{\prime }\left(p_{i}\right)=m\left(p_{i}\right)-w\left(p_{i},t_{j}\right)+w\left(t_{j},p_{i}\right),\;\;\;\forall p_{i}\in P$$

An observation about the dynamic behavior of PPN is that not all states in $R^{n}$ can necessarily be reached from a given initial state $m_{0}$. All the states that can be reached form the set of *reachable states*, whose definition is as follows.

**Definition 5** (reachable states).  *The set of reachable states of PPN* $\Sigma =\left(P,T,A,w,m_{0}\right)$, *represented by* R[Σ], *is the minimal set that satisfies:*

1. $m_{0}\in R\left[\Sigma \right]$;
2. *If* $m_{1}\in R\left[\Sigma \right]$ *and there exists* $t_{j}\in T$ *s.t.* $m_{1}\overset{t_{j}}{⟶}m_{2}$, *then* $m_{2}\in R\left[\Sigma \right]$.

With all the definitions presented above, basic procedures as well as dynamic behaviors of a manufacturing process can be formulated by a PPN. Besides formulating explicit control flow like traditional Petri net model, the generalized PPN model is able to describe detailed implicit data bonded with each sub-process. Such ability is important for both manufacturing process modeling and traceability implementation, and readers will appreciate this advantage later when the traceability solution is described in Section 4 and Section 5.

### 3.2. Basic Patterns of Manufacturing Processes

For complex manufacturing processes in reality, there are several workflow patterns. Most of them have been well summarized in [22], and it has been universally acknowledged that five basic patterns are the most popular and common among them. According to the characteristics of manufacturing systems and the traceability requirement, PPN models of these five basic patterns are presented in this part. Most of the manufacturing processes can be formulated using these five patterns, and other more complex processes could be modeled with these basic blocks using similar methodology.

#### 3.2.1. Sequence Pattern

**Figure 2:**
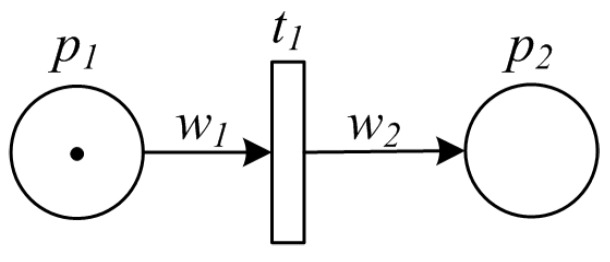
PPN model of sequence pattern.

Sequence pattern means that an activity in a manufacturing process is enabled after the completion of another activity in the same process. Such pattern is the most basic, and examples include production and processing on a single ingredient or intermediate product. A sequence pattern is a “single-input single-output” process, whose PPN model is shown by Figure 2. The implicit data related to the activity—the amount/volume of the item before and after such activity—is indicated by $w_{1}$ and $w_{2}$, respectively. We should note that, it is possible that $w_{1}\neq w_{2}$ due to the processing on the material/item.

#### 3.2.2. Split Pattern

**Figure 3:**
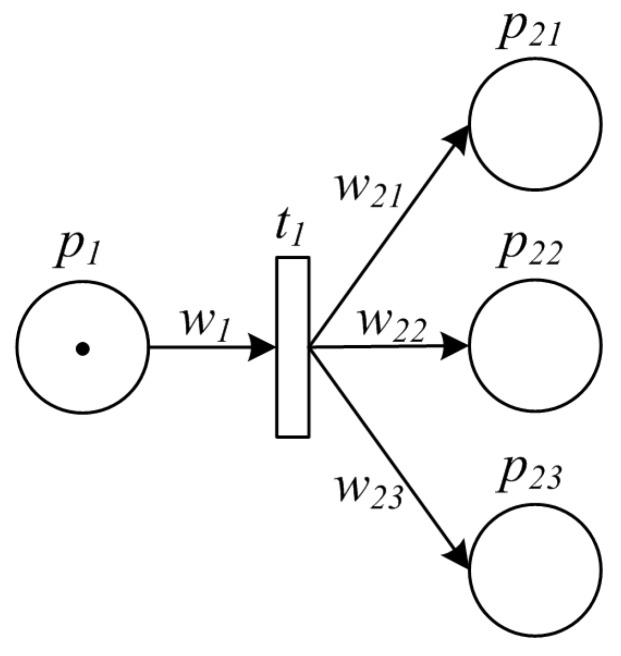
PPN model of split pattern.

Split pattern is a point in the manufacturing process where a single item splits into multiple ones which can be processed in parallel, thus allowing further activities to be executed simultaneously or in any order. An example of split pattern is packaging products into small bags before delivery to the market, and another example is to split a large amount of raw material into small units each of which will be further processed by multiple pipelines. The PPN model for a split pattern is illuminated by Figure 3.

#### 3.2.3. Synchronization Pattern

**Figure 4:**
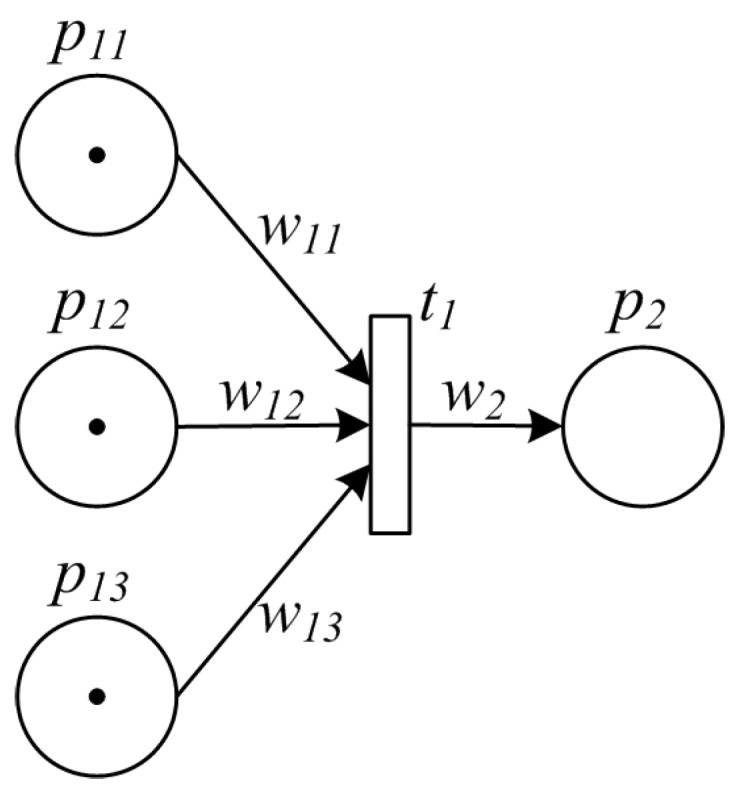
PPN model of synchronization pattern.

In synchronization pattern, multiple items converge into a single one, thus synchronizing multiple activities. One example of such pattern is that middlemen collect materials/goods from small-sized producers, and converge them into a big package for centralized processing. Another example is to integrate multiple ingredients for synthesizing a new item. Figure 4 shows the synchronization pattern formulated by PPN model.

#### 3.2.4. Exclusive Choice Pattern

**Figure 5:**
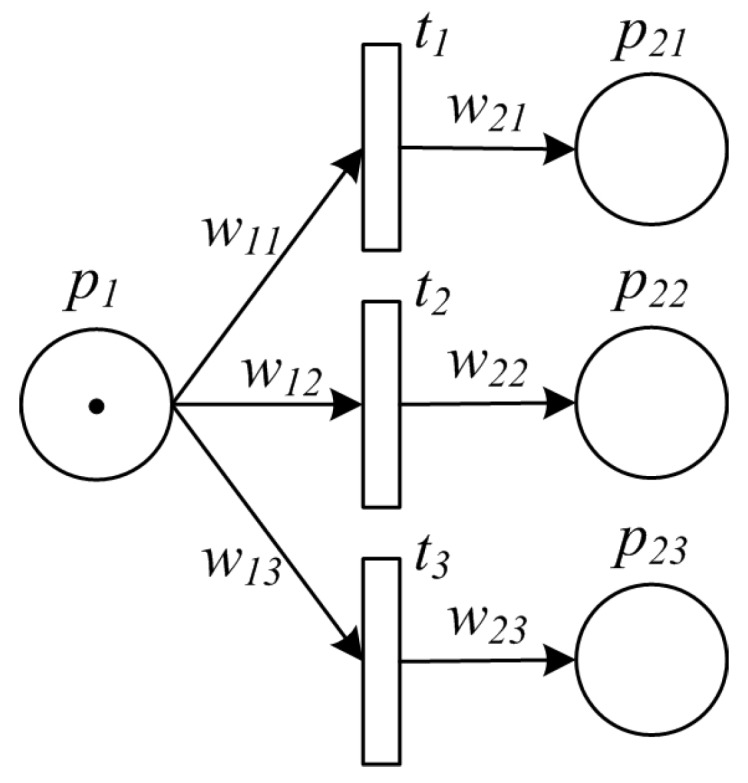
PPN model of exclusive choice pattern.

Exclusive choice pattern indicates the divergence of a branch into two or more branches such that when the incoming task is enabled, the item is immediately passed to precisely one of the outgoing branches based on a mechanism that can select one of the outgoing branches. In reality, the activity of choosing one of the OEMs to produce the required item is a typical example of exclusive choice pattern. In addition, delivering each item to one of the pending markets is another exclusive choice activity in the lifecycle of the products. An intuitive PPN model of exclusive choice pattern is given by Figure 5.

In a traceability solution for manufacturing systems, much more attention has been paid to tracing the liftcycle of the products than the control automata. In order to facilitate traceability analysis which will be introduced in detail in Section 4, the PPN model of exclusive choice pattern is transformed into a split one. With proper data recording in manufacturing process, it can be theoretically proved that the dynamics remain equivalent to the original model after model transformation. Formally, the transformation and its related proofs are given in Theorem 1.

**Theorem 1.**  *Once exclusive choice is determined with definite outcome, its PPN model* $\Sigma =\left(P,T,A,w,m_{0}\right)$ *can be transformed into a split model* $\tilde{\Sigma }=\left(P,\tilde{T},\tilde{A},\tilde{w},m_{0}\right)$ *with the same places and initial state (marking). Suppose the i-th activity is chosen,* ${\tilde{w}}_{1}$ *in the split PPN model is set to be* $w_{1i}$ *of the exclusive choice model,* ${\tilde{w}}_{2i}$ *in the split one to be* $w_{2i}$ *of the exclusive choice pattern while* ${\tilde{w}}_{2j}=0$ *for* ∀j≠i.

**Proof (Proof of Theorem 1).**  In order to prove the soundness of the model transformation, the following two points have to be guaranteed: (1) the dynamics of the state transition remains equivalent; (2) the recorded data related to the activity should be correct.

On one hand, the dynamics issue can be proved by analyzing the state (marking) of the PPN model after the transition. In the original exclusive choice model, given the *i*-th activity is chosen, one can obtain that transition $t_{i}$ fires. According to Definition 4, the marking $m^{\prime }$ of the PPN after $t_{i}$ fires can be expressed as follows.

(1) $$\begin{array}{cc} m^{\prime }\left(p_{1}\right) & =m\left(p_{1}\right)-w_{1i} \end{array}$$

(2) $$\begin{array}{cc} m^{\prime }\left(p_{2i}\right) & =m\left(p_{2i}\right)+w_{2i} \end{array}$$

(3) $$\begin{array}{cc} m^{\prime }\left(p_{2j}\right) & =m(p_{2j}),\;\;\forall j\neq i \end{array}$$

In the transformed split PPN model, $\tilde{m}$ and ${\tilde{m}}^{\prime }$ are supposed to be the markings before and after the transition respectively, and all the model parameters are marked with tildes in order to distinguish the ones in the original exclusive choice model. Since $t_{i}$ is enabled in the original model, we have $\tilde{m}\left(p_{1}\right)=m\left(p_{1}\right)\geq w_{1i}$, and thus ${\tilde{t}}_{1}$ of the split model is enabled. After the firing of ${\tilde{t}}_{1}$, one can obtain that,

(4) $$\begin{array}{cc} {\tilde{m}}^{\prime }\left(p_{1}\right) & =\tilde{m}\left(p_{1}\right)-{\tilde{w}}_{1}=m\left(p_{1}\right)-w_{1i} \end{array}$$

(5) $$\begin{array}{cc} {\tilde{m}}^{\prime }\left(p_{2i}\right) & =\tilde{m}\left(p_{2i}\right)+{\tilde{w}}_{2i}=m\left(p_{2i}\right)+w_{2i} \end{array}$$

(6) $$\begin{array}{cc} {\tilde{m}}^{\prime }\left(p_{2j}\right) & =\tilde{m}\left(p_{2j}\right)+{\tilde{w}}_{2j}=m\left(p_{2j}\right)+0=m\left(p_{2j}\right)\;\;\forall j\neq i \end{array}$$

Therefore, it can be concluded that ${\tilde{m}}^{\prime }=m^{\prime }$.

On the other hand, as $t_{i}$ fires in the original exclusive choice PPN model, the data related to such transition is $w_{1i}$ and $w_{2i}$. In the transformed split model, the nonzero (meaningful) data related to $t_{1}$ is ${\tilde{w}}_{1}=w_{1i}$ and ${\tilde{w}}_{2i}=w_{2i}$. Hence, the correctness of data recording has also been proved. □

#### 3.2.5. Simple Merge Pattern

**Figure 6:**
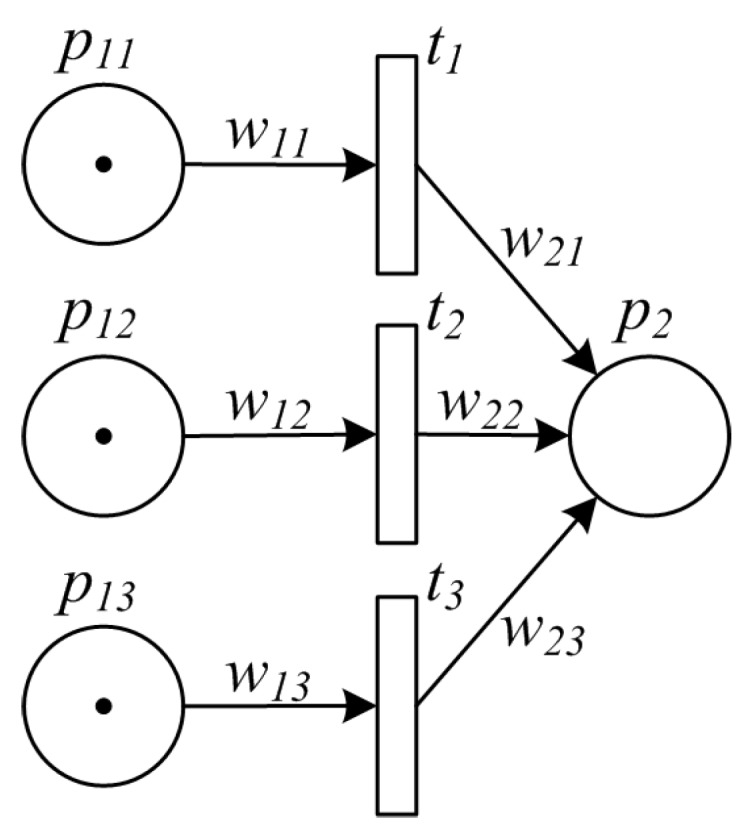
PPN model of simple merge pattern.

For simple merge pattern, two or more alternative branches come together without synchronization, and any one of them can trigger the successor activity. For instance, when there are multiple suppliers for the same raw materials, the provisioning from one of them will immediately start further production activities. Such process pattern can be modeled by the PPN as Figure 6.

Also, a simple merge pattern can be transformed into a synchronization one in a traceability solution. The methodology of model transformation can be found in Theorem 2.

**Theorem 2.**  *Given transition* $t_{i}$ *fires in a simple merge pattern, its PPN model* $\Sigma =\left(P,T,A,w,m_{0}\right)$ *can be transformed into a synchronization model* $\tilde{\Sigma }=\left(P,\tilde{T},\tilde{A},\tilde{w},m_{0}\right)$ *with the same places and initial state (marking).* ${\tilde{w}}_{1i}$ *in the synchronization PPN model is set to be* $w_{1i}$ *of the simple merge model, and* ${\tilde{w}}_{1j}$ *is set to be 0 for* ∀j≠i. ${\tilde{w}}_{2}$ *in* $\tilde{\Sigma }$ *is set to be* $w_{2i}$ *of the original PPN model.*

**Proof (Proof of Theorem 2).**  Similarly, we firstly study the dynamics of the state transitions. Given transition $t_{i}$ fires, we obtain the markings of the original PPN model Σ before and after the firing denoted by *m* and $m^{\prime }$, respectively.

(7) $$\begin{array}{cc} m^{\prime }\left(p_{1}i\right) & =m\left(p_{1i}\right)-w_{1i} \end{array}$$

(8) $$\begin{array}{cc} m^{\prime }\left(p_{1j}\right) & =m(p_{1j}),\;\;\forall j\neq i \end{array}$$

(9) $$\begin{array}{cc} m^{\prime }\left(p_{2}\right) & =m\left(p_{2}\right)+w_{2i} \end{array}$$

In the transformed synchronization PPN model $\tilde{\Sigma }$, the markings before and after the transition are denoted by $\tilde{m}$ and ${\tilde{m}}^{\prime }$, respectively. Given $t_{i}$ is enabled before transition, we have $m\left(p_{1i}\right)\geq w_{1i}$, and thus we know that $\tilde{m}\left(p_{1i}\right)=m\left(p_{1i}\right)\geq w_{1i}={\tilde{w}}_{1i}$. Therefore, ${\tilde{t}}_{1}$ in the transformed model is enabled. After the firing of transition ${\tilde{t}}_{1}$, one can obtain that,

(10) $$\begin{array}{cc} {\tilde{m}}^{\prime }\left(p_{1i}\right) & =\tilde{m}\left(p_{1i}\right)-{\tilde{w}}_{1i}=m\left(p_{1i}\right)-w_{1i} \end{array}$$

(11) $$\begin{array}{cc} {\tilde{m}}^{\prime }\left(p_{1j}\right) & =\tilde{m}\left(p_{1j}\right)-{\tilde{w}}_{1j}=m\left(p_{1j}\right)-0=m\left(p_{1j}\right),\;\;\forall j\neq i \end{array}$$

(12) $$\begin{array}{cc} {\tilde{m}}^{\prime }\left(p_{2}\right) & =\tilde{m}\left(p_{2}\right)+{\tilde{w}}_{2}=m\left(p_{2}\right)+w_{2i} \end{array}$$

Therefore, we have ${\tilde{m}}^{\prime }=m^{\prime }$, and thus prove the equivalence of their dynamics.

Secondly, for all nonzero data related to the manufacturing process, $w_{1i}$ and $w_{2i}$ are recorded in the original PPN model while ${\tilde{w}}_{1i}=w_{1i}$ and ${\tilde{w}}_{2}=w_{2i}$ are recorded in the transformed model. It is clear that the data recorded remains same after the model transformation. □

## 4. Traceability Model and Algorithms

The previous section introduces a PetriNet-based approach for process modeling in an automated manufacturing system. With typical patterns, manufacturing processes can be formulated using uniformed bases. Furthermore, such model can be transformed into a traceability model automatically, which can provide powerful support for tracing the entire life of a product or intermediate item.

### 4.1. Traceability Model

The PPN model formulates the manufacturing process in a forward order from raw materials to final products. For a traceability solution, however, tracing backward from a retail product or an intermediate item to its origin is another important requirement, in order to identify sources of contamination and other potential causes of accidents. To facilitate automatic traceability analysis, *Traceability Petri Net (TPN)* is proposed to formulate a traceability process.

**Definition 6** (Traceability Petri Net).  *A Traceability Petri Net (PPN)* $\hat{\Sigma }$ *for a manufacturing process* $\Sigma \;=\;\left(P,T,A,w,m_{0}\right)$ *is defined by a 5-tuple*

$$\hat{\Sigma }=\left(\hat{P},\hat{T},\hat{A},\hat{w},{\hat{m}}_{0}\right)$$

*where*

- $\hat{P}=T$ *is the finite set of places.*
- $\hat{T}=P$ *is the finite set of transitions.*
- $\hat{A}=\left\{\left(p,t\right)|\left(t,p\right)\in A,t\in T,p\in P\right\}\cup \left\{\left(t,p\right)|\left(p,t\right)\in A,t\in T,p\in P\right\}$ *is the set of arcs from places to transitions and from transitions to places.*
- $\hat{w}:A\to R^{+}\cup \left\{0\right\}$ *is the weight function of the arcs, where* w(p,t)=w(t,p) *for* ∀(t,p)∈A,t∈T,p∈P *and* w(t,p)=w(p,t) *for* ∀(p,t)∈A,t∈T,p∈P.
- ${\hat{m}}_{0}:\hat{P}\cup \hat{T}\to R^{+}\cup \left\{0\right\}$ *is the initial state of the net, indicating the starting point of the tracing process.*

The basic idea of building the transformed TPN model from a PPN is as follows. All the transitions in the original PPN model are transformed into places, while places of PPN are transformed into transitions. The connection relationship of arcs in Σ remains the same, however, their direction are turned into their opposite. The weights are basically unchanged, and the initial state of $\hat{\Sigma }$ indicates starting point where the tracing is originated. Here, the starting point can be either some places of $\hat{\Sigma }$ indicating that the tracing can be started from a manufacturing activities, or some transitions of $\hat{\Sigma }$ which means that one can also start to trace the lifecycle of a intermediate item during the process or a retail product from the market.

The basic process of building the TPN model is to traverse all the places, transitions and arcs of the original PPN, and thus one can obtain that the computational complexity of constructing TPN is O(|P|+|T|+|A|). Therefore, the time-complexity is proportional to the scale of the original PPN.

With Definition 6, the corresponding TPN model of the manufacturing process shown in Example 1 can be constructed. Details of the TPN model can be found as follows.

**Figure 7:**
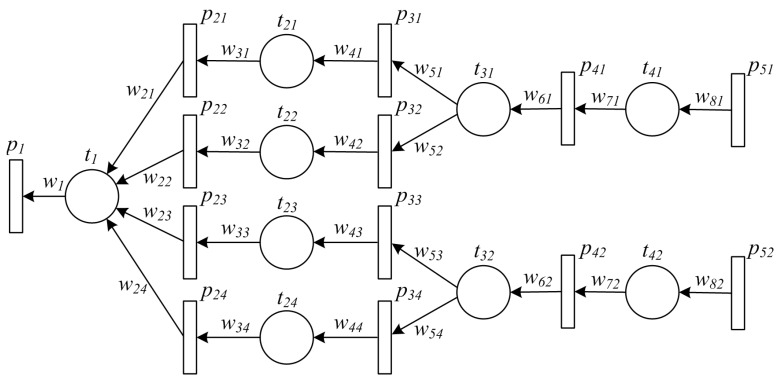
An example of TPN model for a typical manufacturing process.

**Example 2.**  Figure 7 shows a TPN model of the PPN in Example 1.

The definition of *marking* is exactly the same as the one of PPN. Readers may refer to Definition 2 for details. However, each value in a marking has different meaning from PPN, which indicates the proportion of an ingredient in the final product, or the probability that each item has been contaminated. In most cases, the values should be in [0,1]. On the other hand, it should be noted that the definition of the initial state in TPN is different from PPN. Both places and transitions can be defined as one of the initial states, and thus ${\hat{m}}_{0}$ may not be a marking of the TPN. Such different definition provides the TPN model with the ability of tracing backward the manufacturing process from both items and atomic activities. We defer readers to the next subsection for detailed means of dealing with such differences.

In order to facilitate the traceability analysis, we present a different definition of enabled transition in a TPN from that of PPN, shown by Definition 7.

**Definition 7** (enabled transition of TPN).  *A transition* $t_{j}\in \hat{T}$ *in a TPN* $\hat{\Sigma }=\left(\hat{P},\hat{T},\hat{A},\hat{w},{\hat{m}}_{0}\right)$ *is enabled under marking m iff*

$$m\left(p_{i}\right)>0,\;\;\;\text{for}\;\forall p_{i}\in \hat{P},w\left(p_{i},t_{j}\right)>0.$$

The formal definition of firing of an enabled transition in a TPN can be found in Definition 8.

**Definition 8** (firing of TPN).  *When a transition* $t_{j}\in \hat{T}$ *in a TPN* $\hat{\Sigma }=\left(\hat{P},\hat{T},\hat{A},\hat{w},{\hat{m}}_{0}\right)$ *is enabled under marking m, the firing of transition* $t_{j}$ *is denoted by* $m\overset{t_{j}}{⟶}m^{\prime }$ *where*

$$m^{\prime }\left(p_{i}\right)=\left\{\begin{array}{cc} 0 & w(p_{i},t_{j})>0\\ m\left(p_{i}\right)+\frac{w(t_{j},p_{i})}{\sum_{k}w\left(t_{j},p_{k}\right)}\cdot \sum_{l}\frac{w(p_{l},t_{j})}{\sum_{k}w\left(p_{l},t_{k}\right)}m\left(p_{l}\right) & w(t_{j},p_{i})>0\\ m(p_{i}) & \text{otherwise} \end{array}\right.$$

### 4.2. Algorithm for Traceability Analysis

The TPN model presented in the above subsection is able to facilitate the traceability analysis in manufacturing processes. The basic idea is to construct a reachability tree of the TPN. In order to introduce the approach in detail, we first present the basic concept of *reachability tree* of a TPN.

**Definition 9** (reachability tree).  *The reachability tree of a traceability Petri net* $\hat{\Sigma }=\left(\hat{P},\hat{T},\hat{A},\hat{w},{\hat{m}}_{0}\right)$ *is a tree that describes the dynamics of* $\hat{\Sigma }$, *where tree nodes are Petri net states and arcs represent transitions.*

A finite representation of reachability tree can be constructed by an algorithm which will be presented later. To do so, we next introduce some notation as follows.

- *Root node*. Root node is the first node of the reachability tree, obtained from the initial state of the given TPN.
- *Terminal node*. This is any node from which no transition of the TPN can fire.
- *Duplicate node*. This is a node that is identical to a node already in the reachability tree.
- *Node dominance*. Let $m_{1}$ and $m_{2}$ be two markings (states), *i.e.*, nodes in the reachability tree. We say that “$m_{1}$ dominates $m_{2}$”, denoted by $m_{1}{>}_{d}m_{2}$, if the following two conditions hold:
  1. $m_{1}\left(p_{i}\right)\geq m_{2}\left(p_{i}\right)$, for all i=1,2,…,n;
  2. $m_{1}\left(p_{i}\right)>m_{2}\left(p_{i}\right)$, for at least some i=1,2,…,n.
- *Symbol ω*. The symbol *w* in a reachability tree means “infinity” in representing the marking of an unbounded place. For ∀n∈N, we specify n<ω and ω+n=ω-n=ω. It should be noted that, although common in traditional Petri nets, such symbol is rare in TPN, for most of the manufacturing processes are finite and non-iterative. For preciseness, however, such symbol is still introduced in this approach.

With all the notations presented above, the reachability tree of a TPN can be constructed by the traceability algorithm, which is formally presented by Algorithm 1.

The construction procedures of the reachability tree meanwhile are tracing backward from the manufacturing process from the initial state of the TPN. Firstly, all the transitions in ${\hat{m}}_{0}$ are fired, and the initial marking can be obtained which forms the root note of the reachability tree. Next, the algorithm iteratively fires all enabled transitions in a breadth-first order and recursively constructs the reachability tree. The information of the fired transition is pushed into a queue which gathers all the information of semi-finished products that may be contaminated and need to be further examined. Besides the basic information, a weight is pushed into the queue as well, which indicates the probability that each semi-finished product in the manufacturing process being flawed. Finally, once completing the construction process, we may either directly dequeue traceability information in the (reversed) order of the original manufacturing process, or sort the items according to their corresponding weights putting first things first.

The construction of reachability tree has been an open problem in Petri net research for decades. Recently, existing literatures on reachability problem of Petri net have proved that the time complexity of the algorithm can be bounded by $O(N^{|\hat{P}|})$ in worst case [23]. However, in the traceability analysis of a manufacturing process where the activities have been determinate resulting in the finiteness of the manufacturing process, Algorithm 1 presented in this paper can be normally completed in $O(n\cdot |\hat{T}|)$ time where *n* is the number of activities in the process. It should be noted that, although very rarely, it might also be exponential to the number of places of the TPN model in the worst cases.

**Algorithm 1** Traceability Algorithm on TPN**Input:** TPN $\hat{\Sigma }=\left(\hat{P},\hat{T},\hat{A},\hat{w},{\hat{m}}_{0}\right)$**Output:** traceability information *Q*1:Let $m\left(p_{i}\right)\leftarrow {\hat{m}}_{0}\left(p_{i}\right)$ for all $p_{i}\in \hat{P}$.2:**for all**
$t_{j}\in \hat{T}$ and ${\hat{m}}_{0}\left(t_{j}\right)>0$
**do**3: Let $m^{\prime }\left(p_{i}\right)\leftarrow \left\{\begin{array}{cc} m\left(p_{i}\right)+\frac{w(t_{j},p_{i})}{\sum_{k}w\left(t_{j},p_{k}\right)}{\hat{m}}_{0}\left(t_{j}\right) & w\left(t_{j},p_{i}\right)>0;\\ m\left(p_{i}\right) & \text{otherwise}. \end{array}\right.$4: Let $m\leftarrow m^{\prime }$.5:**end for**6:Initialize *m* as the root node of reachability tree. Let Ψ←{m}.7:**for all**
ψ∈Ψ
**do**8: **if** no transition is enabled at state (under marking) *ψ*
**then**9:  *ψ* is a terminal node.10: **else**11:  Create a new node $\psi^{\prime }$ s.t. $\psi \overset{t_{j}}{⟶}\psi^{\prime }$ for some $t_{j}\in \hat{T}$.12:  $Q.\text{enqueue}(t_{j},\sum_{l}\frac{w(p_{l},t_{j})}{\sum_{k}w\left(p_{l},t_{k}\right)}m\left(p_{l}\right))$.13:  **if**
$\psi (p_{i})=\omega$ for some $p_{i}\in \hat{P}$
**then**14:   Set $\psi^{\prime }\left(p_{i}\right)\leftarrow \omega$.15:  **end if**16:  **if** there exists a node *θ* such that $\psi^{\prime }{>}_{d}\theta$
**then**17:   Set $\psi^{\prime }\left(p_{i}\right)\leftarrow \omega$ for all $p_{i}\in \hat{P}$ s.t. $\psi^{\prime }\left(p_{i}\right)>\theta \left(p_{i}\right)$.18:  **end if**19:  Let $\Psi \leftarrow \Psi \cup \{\psi^{\prime }\}$20: **end if**21: Let Ψ←Ψ-{ψ}.22:**end for**

## 5. Prototype System

In this section, a practical approach of traceability solution in manufacturing systems is introduced. Detailed designs, techniques, as well as a case study in reality are presented.

### 5.1. Framework

**Figure 8:**
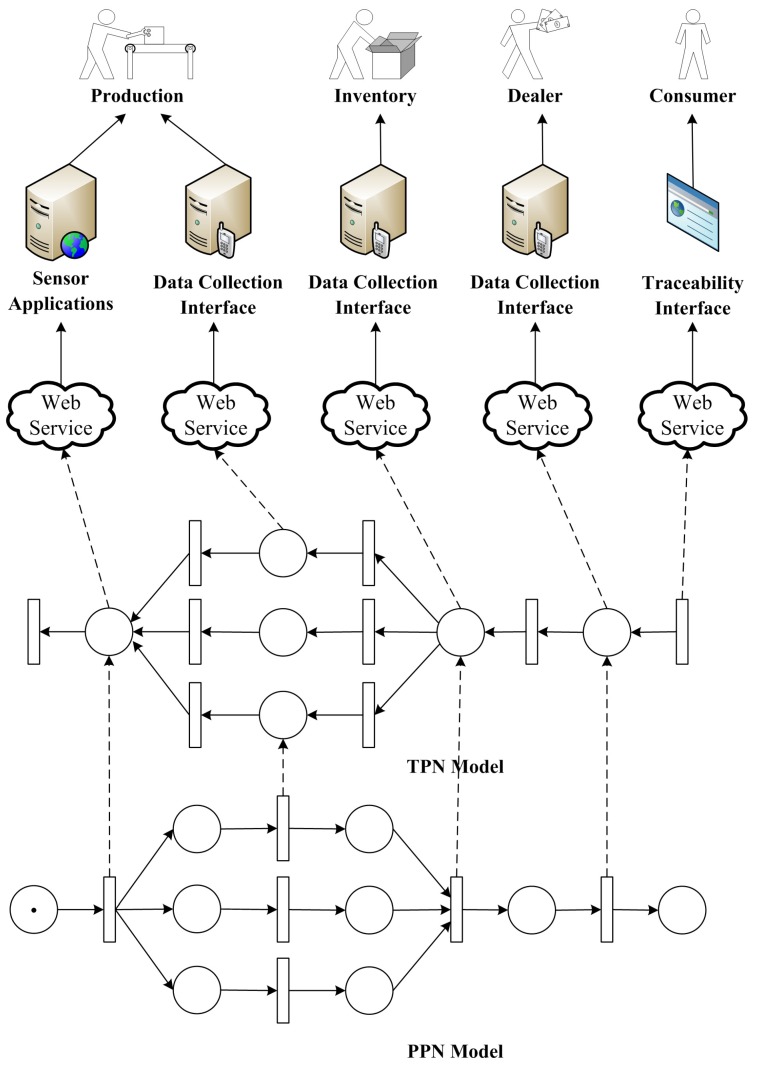
Framework of a traceability solution for cyber-physical manufacturing systems.

Figure 8 shows an overall framework of the approach for supporting traceability in cyber-physical manufacturing systems. At the very beginning, a manufacturing process is formulated by a PPN describing detailed activities in the whole lifecycle of the product from raw materials to final market, and thus the manufacturing process can be guided by the model. During the productions, atomic activities are encapsulated by web services, which provide precise interfaces for collecting data that enable the traceability process. Once a request for traceability analysis is submitted by either a consumer or a producer, the corresponding TPN model is build and traceability algorithm is conducted.

The approach presented in this paper is able to trace the whole life of a product. Besides traditional manufacturing process, transportation, inventory as well as delivery can be tracked, since most of them follow the basic patterns of manufacturing processes and thus can be well modeled by PPN. With modern CPS techniques, some of the activities can be accomplished fully automatically, and data can be collected by sensors attached to the manufacturing equipments. For others which involve human activities and lack of sensors, data collection interfaces are provided via web services for manual input. In specific, such data input procedure can be accomplished with either manually keyboard typing, speech recognition, barcode scanners, or RFID readers.

### 5.2. Technical Implementation

With the rapid development of services computing technology [24], web service has been widely applied in many IT-based systems. It provides programmable modules with standard interface descriptions that enable universal accessibility through standard communication protocols. It encapsulates heterogeneous functionalities of services, and can be universally monitored, discovered, invoked, and controlled through Uniform Resource Identifier (URI). Due to its powerful ability in dealing with heterogeneity, web service technology is applied into this approach to encapsulate atomic activities in manufacturing processes.

**Figure 9:**
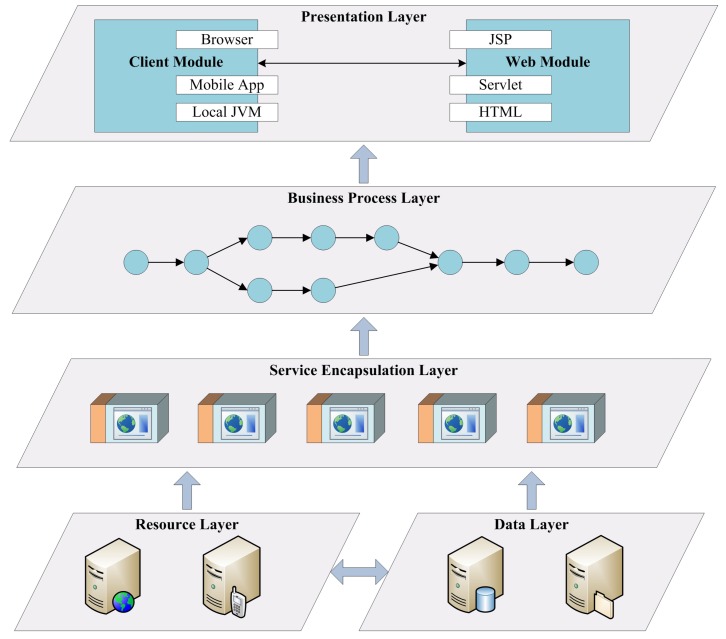
Framework of web service encapsulation.

Figure 9 shows a basic framework of a web service based system. Devices especially sensors in manufacturing processes are encapsulated by a uniform devices abstraction approach based on Open Service Gateway Initiative (OSGi) technology [25], and heterogeneous data and device operations can be accessible with a uniform web service interface. It should be noted that, the details about the control on manufacturing devices are out of the scope of this paper which mainly seeks to solve the traceability problem. However, if the device control provides a web service interface, it can be easily embedded into the manufacturing process; otherwise, it may be treated as a legacy system and wrapped into a uniform service interface with some middleware adaptor techniques such as IBM WebSphere Business Integration Adaptor [26] and Jave EE Connector.

In order to facilitate traceability analysis, database servers are deployed in this prototype system to record information related to manufacturing processes. According to the characteristics of basic patterns of manufacturing activity that have been presented in Section 3, an automatic data entity generation scheme is designed in order to improve the efficiency of system development and avoid human errors in service design and programming. In specific, basic data should include IDs and weights of items before and after the activities, and some of them can be obtained or generated automatically by the system. For example, the IDs of items before an activity can be obtained by barcode or RFID readers, and the weights/amounts before and after the activity can be obtained by measurement equipments. Furthermore, the item IDs after the activity can be automatically generated following certain encoding rules, and RFID tags or barcode labels can be produced with generated information. More specifically, the data entities for different manufacturing process patterns are formulated according to their traceability requirements, shown as Table 1.

Besides the basic traceability information presented above, other data related to each manufacturing activity throughout the supply chain should also be recorded. Since such data is closely related to different activities and varies significantly, we leave it for domain developers to implement data collection interfaces and design database tables when realizing specific web services for manufacturing activities. This variety can be partly illustrated later when we describe a case study in Section 5.3.

A manufacturing process may consist of a collection of atomic activities, each of which is encapsulated by a web service for automatic control and traceability data collection. In this approach, the process is practically formulated by Web Services Business Process Execution Language (WS-BPEL), which is an OASIS standard executable language for specifying actions within business processes with web services. Using such formulation, composite services can be executed and managed by BPEL-supported workflow engines. The transformation between Petri Net and WS-BPEL has been studied for years, which has been presented in several existing approaches for service composition analysis [27].

Finally, the services are provisioned based on a browser/server (B/S) architecture, and users can invoke the services through JSP or HTML web pages on their browsers. Also, in order to support various mobile devices especially embedded CPS equipments, customized mobile applications can also invoke the services for supporting traceability of manufacturing processes.

### 5.3. Case Study

The traceability approach presented in this paper has been applied to a quality control system for bee products (including honey, propolis, royal jelly, *etc.*). Such system monitors the whole manufacturing process and traces all the information during the lifecycle of every product, in order to improve the safety of bee products. It has been widely deployed in China, covering more than 113,600 bee colonies in 13 provinces.

The manufacturing process of bee products basically consists of the 5 following steps. (1) Raw materials are raised and harvested by farmers from farms, and they are labeled according to their beehives or colonies; (2) The brokers then evaluate the quality of the raw materials, purchase them from farmers, and resell them to the factories. In such step, some materials form different beehives/colonies might be merged together into bigger bottles; (3) Once finishing collecting raw materials, factories process them by melting, purification, and sterilization; (4) After the processing, edible products are packaged into small bottles/boxes for sale; (5) Finally, they are delivered to retail on the market.

**Figure 10:**
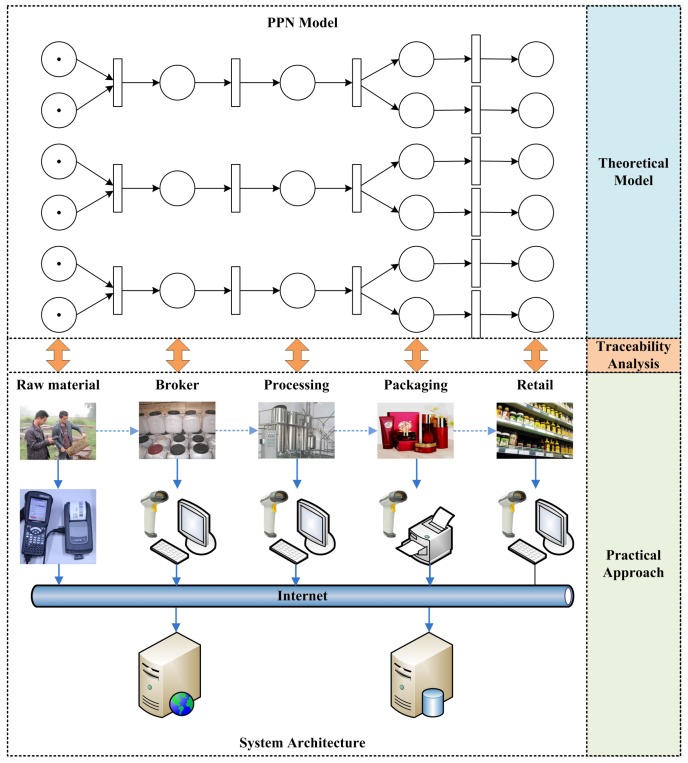
Framework of quality control system for bee products.

Figure 10 shows both theoretical and practical frameworks of the system. According to the procedures of the bee product manufacture presented above, the PPN model of the manufacturing process is first constructed. Raw materials are the starting points of the process, regarded as initial states of the PPN model. Brokers collect raw materials from different origins and put them together into bigger bottles, and thus this activity can be modeled following the typical synchronization pattern. The processing and delivery are simply two sequence activities, and the packaging conforms to a split pattern.

For the practical part of the system, we design and develop applications on both mobile device and PC for automatically collecting manufacturing data to facilitate traceability analysis. Each farmer is equipped with a PDA and a portable barcode printer when harvesting raw materials. Basic information on the materials and farms are collected by either voice input or manual typewriting, and traceable barcodes are automatically generated using the symbology of GS1-128 (formerly known as UCC/EAN-128). Once the information has been confirmed, an adhesive barcode label is automatically printed by the portable printer attached with the PDA (see Figure 10), and is stickered on the bottle of raw materials. On the other hand, most of the other activities are processed indoor, and PCs with Internet connection are used for data collection. Each PC is equipped with a USB barcode scanner which enables fast label reading, and a label printer which produces barcode labels adhered to semi-finished products.

All the data collection activities are handled through a uniform web service interface, and their underlying data is stored in a database server. In Figure 10, the solid arrows represent the data flows in the manufacturing process, while the dashed ones show the control flows.

**Figure 11:**
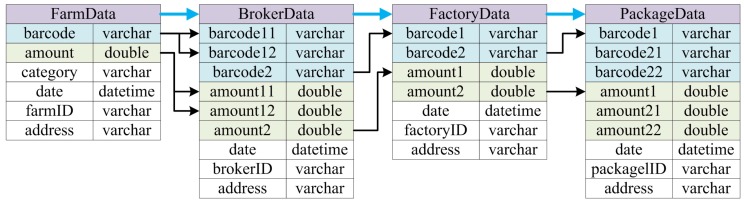
Data entities of quality control system for bee products.

More specifically, the underlying data entities are shown by Figure 11. Blue arrows indicate the control flow of the manufacturing process, while the black ones represent the data flow. According to the PPN model, some of the data entities can be automatically generated, indicated by colored ones in the figure. They include the barcodes of the items before and after each activity which are blue-colored, and their corresponding amounts shown in green. Other data entities are designed by system developers according to the characteristics of the manufacturing activities, such as the ID and address of each participant, the date and time of each activity, and the category of the raw materials harvested by farmers (*i.e.*, Chinese date honey, sunflower honey, *etc.*).

**Figure 12:**
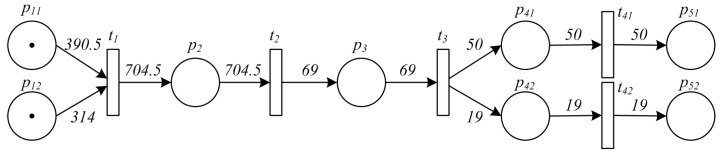
PPN model of the manufacturing process of an orange honey product.

**Figure 13:**
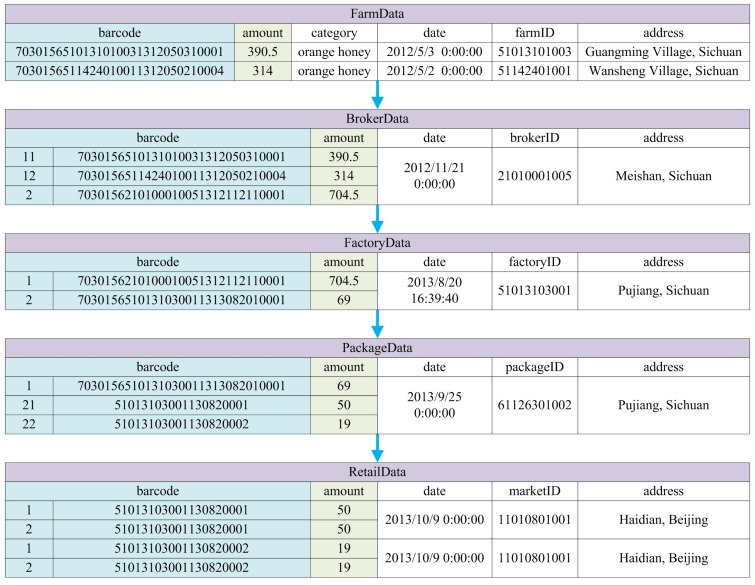
Original data of the manufacturing process of an orange honey product.

**Figure 14:**
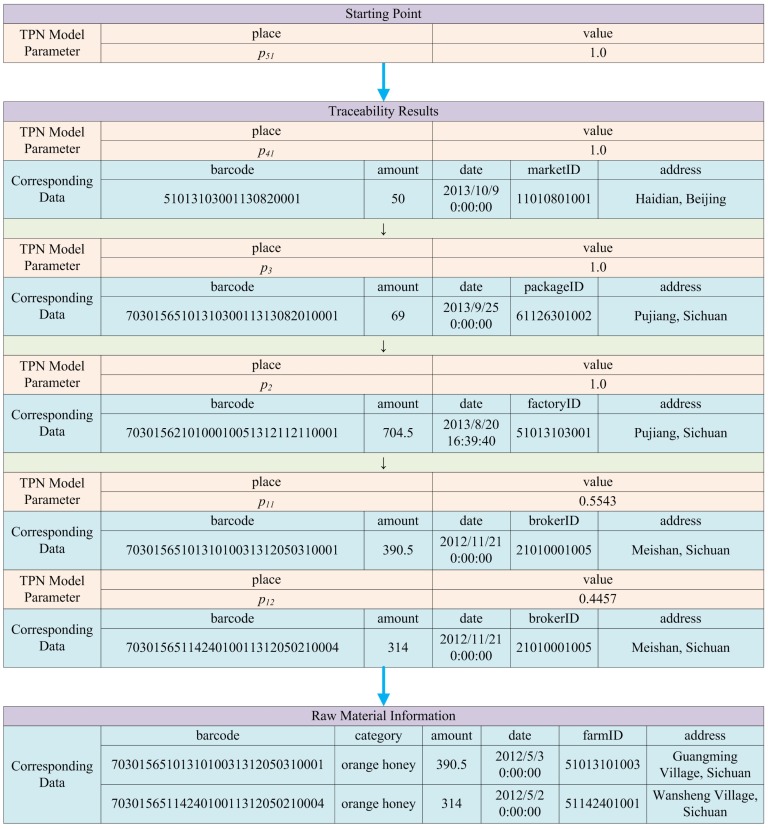
Results of traceability analysis of an orange honey product.

In order to illuminate the effectiveness of the approach, an example from reality is presented, showing the traceability process of an orange honey item. The raw materials are collected from two different farms in Sichuan Province (in China) before being merged together for processing, and they are finally divided into two different sized bottles for retail. All the data related to each of the activity in such manufacturing process has been precisely recorded, and traceability analysis from a retail item is conducted using the approach proposed in this paper. Figure 12 shows the original PPN model of the manufacturing process, and hence Figure 13 and Figure 14 show the original recorded data and the result of the analysis, respectively.

Specifically, the analytical results obtained by the algorithm are shown in red, indicating every place and its corresponding value (proportion of ingredient or probability of contamination) in the process of traceability analysis. Based on them, production data can be found from the database according to the barcodes, and detailed information is displayed to users which is shown by the blue tables in Figure 14.

## 6. Conclusions

Traceability has always been an important issue in manufacturing, especially for food and clinical materials. This paper proposes a theoretical approach for traceability analysis of CPS-based manufacturing systems. The Petri net model is generalized to formulate a manufacturing process, and a scheme for obtaining its corresponding traceability model is presented. An algorithm is proposed for traceability analysis, based on which a practical prototype system is designed. The approach presented in this paper has been successfully applied to a real-life quality control system for bee products, which has been widely deployed in China. It is expected to provide both theoretical and practical reference value for the design and development of modern automatic manufacturing systems.

There are several avenues for future work. Firstly, the traceability problem is important not only to manufacturing but also to other systems like healthcare and transportation where CPS technologies have also been widely applied for enhanced robustness and efficiency [28]. The basic idea of the modeling approach proposed in this paper may have potential contribution to other domains by theoretically formulating related fundamental activities and conducting traceability analyses. Secondly, time is an essential issue in CPS. Although the time points at which manufacturing activities are enabled are recorded in the database for potential traceability analysis in this approach, such issue can be further studied theoretically. Time can be precisely modeled and formulated by some generalized Petri net models, e.g., Timed Petri Net, Stochastic Petri Net, *etc.*, by further equipping the Petri Net model with a clock structure, which can be valuable especially for time series analysis and dynamic optimal control. Thirdly, the performance and trustworthiness issues can be further addressed in the future. Proper trust management, fault tolerance and performance optimization on system components, sensing data and underlying networking infrastructure are of value for the traceability analysis or even the whole cyber-physical manufacturing system.

## Figures and Tables

**Table 1:** Data entities of basic manufacturing process patterns.

	Sequence Pattern	Split Pattern	Synchronization Pattern
Data obtained by reader/scaner	$id_{1}$	$id_{1}$	$id_{11},id_{12},\ldots ,id_{1n}$
Data obtained by measurement	$w_{1},w_{2}$	$w_{1},w_{21},w_{22},\ldots ,w_{2n}$	$w_{11},w_{12},\ldots ,w_{1n},w_{2}$
Automatically generated data	$id_{2}$	$id_{21},id_{22},\ldots ,id_{2n}$	$id_{2}$
